# Artificial intelligence and biomarker-driven prediction of post–coronary artery bypass grafting atrial fibrillation: Integrating clinical, genomic, and metabolic insights

**DOI:** 10.1016/j.hroo.2026.06.006

**Published:** 2026-06-18

**Authors:** Muhammad Usman Ghani, Abhishek Prasad, Jai Sivanandan Nagarajan, Darsh Tusharbhai Patel, Rupak Desai, Subramanian Gnanaguruparan, Subhasis Chatterjee

**Affiliations:** 1 Department of Internal Medicine, Central Michigan University, Mt. Pleasant, Michigan; 2 Department of Anesthesiology and Perioperative Medicine, MD Anderson Cancer Center, Houston, Texas; 3 Department of Medicine, SUNY Upstate Medical University, Syracuse, New York; 4 Department of Medicine, Mercy Catholic Medical Center, Darby, Pennsylvania; 5 Independent Researcher, Outcomes Research, Atlanta, Georgia; 6 Division of Cardiology, Christiana Hospital, Newark, Delaware; 7 Michael E. DeBakey Department of Surgery, Baylor College of Medicine, Houston, Texas

**Keywords:** Artificial intelligence, Machine learning, Postoperative atrial fibrillation, Coronary artery bypass grafting, Predictive modeling

## Abstract

**Background:**

Postoperative atrial fibrillation (POAF) remains one of the most frequent and consequential complications after coronary artery bypass grafting, occurring in 15%–30% of patients and contributing to increased morbidity, prolonged hospitalization, and higher long-term mortality. Despite decades of investigation, traditional risk models have shown limited predictive accuracy owing to the multifactorial nature of POAF.

**Objective:**

This review synthesizes the emerging evidence from recent studies applying artificial intelligence (AI) and machine learning (ML) approaches for POAF prediction, with focus on clinical, biochemical, genomic, and molecular dimensions.

**Methods:**

We conducted a structured narrative review of PubMed/MEDLINE, Embase, and Google Scholar for studies evaluating AI and ML-based prediction of POAF following isolated coronary artery bypass grafting published from January 2020 through 2025. Search terms included POAF, coronary artery bypass grafting, ML, AI, pharmacogenomics, and biomarkers. A total of 9 studies met inclusion criteria and were synthesized narratively given the heterogeneity in study designs and outcomes.

**Results:**

Across studies, modern algorithms demonstrate areas under the curve receiver operating characteristic between 0.80 and 0.93, with performance exceeding that reported for traditional clinical risk scores. However, external validation, model calibration, and biases remain to be fully addressed before clinical translation is considered.

**Conclusion:**

The convergence of interpretable AI, metabolic and genetic biomarkers, and clinical data offers a promising path toward individualized risk stratification and targeted postoperative management.

**Key Findings**

▪ Across recent studies, machine learning models built from routine perioperative data achieve areas under the receiver operating characteristic curve (AUCs) ranging from 0.80 to 0.93 for predicting postoperative atrial fibrillation (POAF) after coronary artery bypass grafting, with performance generally exceeding traditional risk scores, such as CHA_2_DS_2_-VASc, HATCH, and the POAF score (typical AUCs 0.60–0.70).
▪ The highest-performing models typically integrate multiple algorithmic approaches and are derived from larger cohorts, with explainable artificial intelligence techniques consistently identifying key predictors, including age, atrial structural remodeling, renal function, operative duration, and systemic inflammation.
▪ Biomarker- and omics-driven approaches, spanning pharmacogenomic, metabolomic, stress-metabolic, and microRNA signatures, show biologically coherent signal but remain exploratory, often constrained by small samples, single-center designs, and limited external validation.
▪ The collective evidence suggests a hierarchical risk architecture in which clinical and structural variables carry most predictive weight, with molecular features providing incremental value; however, limited external validation, underreporting of calibration, and lack of clinical utility assessment currently limit translation into routine practice.

## Introduction

Postoperative atrial fibrillation (POAF) is the most common arrhythmia occurring after coronary artery bypass grafting (CABG), affecting approximately 15%–30% of patients undergoing this procedure.^1^, ^2^, ^3^, ^4^ The development of POAF is associated with increased risk of stroke, heart failure, infection, prolonged hospitalization, and higher long-term mortality.^1^, ^2^, ^3^ Although POAF may resolve spontaneously in many patients, it frequently represents a clinically significant postoperative complication requiring additional monitoring and therapeutic intervention.

Current prevention strategies include perioperative β-blockers and amiodarone in combination with rhythm surveillance.^5^ Contemporary management has evolved toward a multimodal strategy incorporating preoperative pharmacologic prophylaxis, intraoperative approaches such as posterior pericardiotomy, and individualized postoperative rhythm and anticoagulation management.^6^ Despite these advances, existing clinical risk scores, including CHA_2_DS_2_-VASc, HATCH, EuroSCORE-based models, and the POAF score, demonstrate only modest predictive performance, with areas under the receiver operating characteristic curve (AUCs) typically ranging from 0.60 to 0.70.^7^, ^8^, ^9^, ^10^

Traditional models rely primarily on demographic and perioperative clinical variables and often fail to capture the complex metabolic, inflammatory, and genetic contributors to POAF. As a result, interest has grown in the application of artificial intelligence (AI), specifically machine learning (ML), approaches capable of integrating multidimensional data sources to improve prediction of postoperative arrhythmias. In this review, we summarize and synthesize emerging evidence on AI-based and biomarker-driven prediction models for POAF after CABG, with emphasis on clinical, metabolic, genomic, and electrocardiographic predictors. We confined this review to isolated CABG to preserve methodological homogeneity across the included studies. Specifically, valve and combined cardiac surgery differ from isolated CABG in cardiopulmonary bypass duration, extent of atrial manipulation, and baseline arrhythmic substrate, which may contribute to differences in POAF incidence and limit direct generalization of prediction models developed in isolated CABG cohorts.^3^, ^4^, ^5^, ^6^ Aggregating these populations may therefore obscure rather than clarify the performance of the prediction models under review. In addition, we examine the potential implications of these models for clinical decision making and highlight key limitations related to external validation, calibration, and bias.

## Methods

We conducted a structured narrative review of literature, adhering to the accepted reported standards for narrative reviews. Databases searched included PubMed/MEDLINE, Embase, and Google Scholar, to identify studies evaluating AI-based prediction of atrial fibrillation after isolated CABG. Search terms included “postoperative atrial fibrillation,” “coronary artery bypass grafting,” “machine learning,” “artificial intelligence,” “pharmacogenomics,” and “biomarkers” combined using Boolean operators. We selected clinically relevant studies from January 2020 through 2025 on the basis of methodological rigor, with emphasis on evaluating predictive models incorporating clinical, biochemical, electrographic, genomic, and other molecular features. Studies before this period were identified through manual review of reference lists to provide contextual and baseline comparisons. Findings were synthesized narratively, given heterogeneity in study designs and outcomes. A total of 9 studies met inclusion criteria and were included in the primary synthesis. Given the heterogeneity in study designs, a formal systematic review framework was not applied.

### AI and predictive models based on clinical characteristics

This section summarizes and synthesizes key studies investigating the application of ML approaches in POAF risk assessment. Commonly used approaches identified across all studies include Random Forest (a tree-based ensemble method that combines multiple decision trees to improve prediction), XGBoost (a gradient-boosting algorithm that builds models sequentially), Gaussian Naive Bayes (a probabilistic classifier estimating the likelihood of outcomes based on input features), and stacking ensembles (which combine predictions from multiple models). SHAP (SHapley Additive exPlanations) is used to interpret model predictions by quantifying the contribution of each feature to the final prediction.

CABG procedures expose the myocardium to surgical trauma, ischemia-reperfusion injury, and systemic inflammatory responses that promote atrial electrical and structural remodeling. These physiologic changes create a substrate for atrial arrhythmogenesis during the perioperative period.^3^^,^^4^^,^^6^

Recent advances in AI and ML have enabled integration of large perioperative data sets to identify complex nonlinear relationships among clinical variables. Using these techniques, investigators have developed predictive models for POAF with strong discrimination, with select models reporting AUC values exceeding 0.90,^11^ and others demonstrating improved performance compared with traditional regression-based risk scores.^12^, ^13^, ^14^, ^15^, ^16^ However, generalization of these performance metrics remains limited, as most models have been developed and validated retrospectively within internally validated cohorts.

These models incorporate a range of predictors including demographic characteristics, comorbid conditions, perioperative variables, and laboratory markers. In addition, pharmacogenomic and metabolomic data have been increasingly integrated into predictive algorithms while deep learning techniques have been applied to electrocardiographic signals to detect subtle patterns of atrial vulnerability that may not be visible through conventional analysis.^11^, ^12^, ^13^, ^14^, ^15^, ^16^, ^17^, ^18^

However, many of these models have been derived from single-center cohorts or geographically homogeneous populations. External validation across diverse health care systems remains limited, and the reported performance metrics may therefore overestimate their real-world predictive capability.

Early ML-based investigations demonstrated that decision tree models incorporating patient age, atrial size, ischemia time, and comorbidity burden could modestly predict POAF risk after CABG.^10^ These models showed limited discrimination, with AUC values typically ranging between approximately 0.60 and 0.70, similar to conventional regression-based risk models.

More recent AI-based approaches have substantially improved predictive performance. In an analysis of 2994 CABG procedures, Ma et al^11^ developed an ensemble ML model integrating 11 base learners, achieving an AUC of 0.931 and an F1 score of 0.797 in an external validation cohort, although both derivation and validation cohorts originated from hospitals within the same geographic region, which may limit broader generalizability. The model demonstrated improved discrimination compared with commonly used clinical risk scores, including CHA_2_DS_2_-VASc (AUC 0.713), HATCH (AUC 0.708), and the POAF score (AUC 0.667). Important predictors included brain natriuretic peptide levels, left ventricular end-diastolic diameter, body mass index, perioperative β-blocker use, and operative duration, aligning with established clinical determinants of POAF.

Similarly, Peng et al^12^ applied an XGBoost algorithm to the Comprehensive Observational Study on the Determinants of Postoperative Atrial Fibrillation in Patients Undergoing Coronary Artery Bypass Grafting (CODA-AF CABG) cohort consisting of 13,313 patients and developed a 7-variable inflammatory risk model that achieved AUCs of 0.894 and 0.896 in derivation and validation cohorts, respectively. The model demonstrated strong calibration and decision curve performance, indicating that nonlinear modeling of routine perioperative variables can achieve predictive accuracy comparable to models incorporating specialized biomarkers.

In another cohort of 394 patients undergoing CABG, Parise et al^13^ compared multiple ML approaches, including Random Forest, multivariate adaptive regression splines, neural networks, and support vector machines, for POAF prediction. Random Forest models demonstrated the most reliable performance and identified age, preoperative creatinine levels, aortic cross-clamp duration, and EuroSCORE as key predictors of postoperative arrhythmia risk.

Across studies, explainable AI techniques such as SHAP have increasingly been used to interpret model predictions and identify clinically meaningful drivers of POAF risk.^11^, ^12^, ^13^ Key predictors commonly identified across models include age, atrial and ventricular structural remodeling, renal dysfunction, ischemic or bypass duration, and markers of systemic inflammation.

Collectively, these study results should be interpreted as improved discrimination rather than superior clinical utility. Representative AI models for POAF prediction after CABG and their key characteristics are summarized in Table 1.^11^, ^12^, ^13^^,^^14^^,^^16^^,^^17^^,^^19^, ^20^, ^21^

**Table 1:** Summary of studies investigating AI, ML, and biomarker-based prediction of NOAF/POAF after CABG

Study	Population/design	Model/method	Key predictors/features	Performance (AUC or accuracy)	Principal findings/interpretation
Cao et al^19^	3498 patients undergoing CABG (MIMIC-IV database, retrospective); 80:20 internal training to validation split	XGBoost (best performing among 15 ML models tested)	SHR, age, BUN, po_2_, hemoglobin, systolic blood pressure, BMI, and β-blocker use	Validation cohort: AUC: 0.813 (95% CI 0.772–0.849); accuracy: 0.756 (decreased to 0.779 without SHR)	SHR independently predicted NOAF (OR 1.39; 95% CI 1.04–1.85). SHAP analysis demonstrated SHR, advanced age, and elevated BUN among top contributors to POAF risk
Hua et al^14^	576 patients undergoing CABG with pharmacogenetic testing; external validation in a cohort of 61 patients	Gaussian Naive Bayes (best performing among 8 ML models tested)	Multivessel CABG (≥3 vessels), heart failure history, pharmacogenomic rs5219 (*KCNJ11*), bypass duration	AUC: 0.81 (95% CI 0.70–0.91); accuracy: 0.81 Independent validation cohort: accuracy: 0.79	Integrating pharmacogenomics with clinical features improved POAF risk prediction; developed as a Web-based clinical decision-support risk tool
Akbulut et al^20^	100 patients undergoing CABG (50 POAF vs 50 non-POAF); internal retrospective pilot study without external validation	ML classification	Preoperative Mg, albumin, TIBC	Not applicable (model used primarily for threshold identification and feature ranking rather than for predictive performance scoring)	Identified preoperative laboratory thresholds for POAF risk; Mg <2.0 mg/dL, TIBC >442 μg/dL, albumin <29 g/L. Proof-of-concept application of ML for biomarker-driven prediction
Sathipati et al^21^	15 patients undergoing CABG (7 POAF cases vs 8 non-POAF controls); prospective pilot study without external validation	Random Forest, XGBoost, SVM, K-Nearest Neighbors	Preoperative circulating microRNAs (top 10 selected from 84 candidates), including miR-96-5p, miR-184, miR-17-3p, and miR-200-3p	XGBoost AUC: 0.83; Random Forest AUC: 0.76	POAF miRNA signature linked to cardiovascular pathways (MAPK, PI3K-Akt, and TGF-β); potential molecular predictor of POAF
Ma et al^11^	2994 patients undergoing CABG from 2 tertiary hospitals in China Retrospective derivation cohort (N = 2486) with external validation cohort (N = 508)	Stacking ensemble (11 base ML learners)	BNP, LVEDD, EF, BMI, β-blocker use, age, surgery duration, NPAR, and LA diameter	Derivation cohort: AUC: 0.941; accuracy: 0.917 Validation cohort: AUC: 0.931; accuracy: 0.826 (95% CIs not reported)	AI stacking tool demonstrated improved discrimination compared with traditional risk scores such as CHA_2_DS_2_-VASc, HATCH, and POAF scores; external validation within a single geographic region; model deployed as an easy-to-use bedside risk assessment tool with interpretable SHAP output
Parise et al^13^	394 patients undergoing CABG (retrospective cohort); 75:25 internal train to test split	Random Forest (compared alongside NN, MARS, and SVM)	Age, preoperative creatinine, cross-clamp time, BSA, EuroSCORE	Random Forest: maximum test AUC: 0.78; test accuracy: 0.78 (95% CI 0.69–0.86)	While other models (SVM and NN) achieved higher maximum AUCs, Random Forest was the most robust model, achieving maximum values for all other parameters (including accuracy and precision), emphasizing age, renal function, and cross-clamp duration as predictors of POAF
Peng et al^12^	13,313 patients undergoing CABG (CODA-AF single-center retrospective cohort; 70:30 internal derivation to validation split)	XGBoost (7-variable inflammatory risk model)	Top 7 features via SHAP: lymphocytes, monocytes, eosinophils, leukocytes (white blood cells), C-reactive protein, age, LA anteroposterior diameter	Derivation cohort: AUC: 0.893 (95% CI 0.875–0.913) Validation cohort: AUC: 0.896 (95% CI 0.879–0.914)	Interpretable inflammatory risk model driven by ML, achieving excellent discrimination and calibration. Suggests that routine perioperative inflammatory and clinical data may reliably predict POAF without specialized assays
Yang et al^17^	Patients undergoing CABG with pericardial fluid and serum metabolomics; discovery and internal validation cohort design	ML (Random Forest) + regression	4-metabolite model (aceglutamide, ornithine, methionine, and arginine)	Discovery cohort: AUC: 0.954; accuracy: 93.3% Validation cohort: AUC: 0.872; accuracy: 90.4%	4-metabolite signature accurately differentiated POAF and non-POAF via oxidative stress alterations in cardiac microenvironment; integrated pericardial and serum biomarkers highlight biochemical mechanisms
Folla et al^16^	105 patients undergoing CABG	CART decision tree	LA diameter, age	–	AF more likely with LA diameter >40.5 mm and age >64.5 y; early ML precursor study

AF = atrial fibrillation; AI = artificial intelligence; AUC = area under the receiver operating characteristic curve; BMI = body mass index; BNP = brain natriuretic peptide; BSA = body surface area; BUN = blood urea nitrogen; CABG = coronary artery bypass grafting; CART = classification and regression tree; CI = confidence interval; CODA-AF = Comprehensive Observational Study on the Determinants of Postoperative Atrial Fibrillation in Patients Undergoing Coronary Artery Bypass Grafting; EF = ejection fraction; LA = left atrial; LVEDD = left ventricular end-diastolic diameter; MAPK = mitogen-activated protein kinase; MARS = multivariate adaptive regression spline; MIMIC-IV = Medical Information Mart for Intensive Care IV; ML = machine learning; NPAR = neutrophil percentage-to-albumin ratio; NN = neural network; NOAF = new-onset atrial fibrillation; OR = odds ratio; PI3K-Akt = phosphatidylinositol 3-kinase/protein kinase B pathway; po_2_ = partial pressure of oxygen; POAF = postoperative atrial fibrillation; rs5219 (*KCNJ11*) = potassium inwardly rectifying channel subfamily J member 11 gene variant; SHAP = SHapley Additive exPlanations; SHR = stress hyperglycemia ratio; SVM = Support Vector Machine; TGF-β = transforming growth factor β; TIBC = total iron-binding capacity.

Beyond clinical predictors, pharmacogenomic and genetic factors may further refine POAF risk estimation by capturing interindividual variation in drug metabolism and electrophysiologic susceptibility. Hua et al^14^ applied a Gaussian Naive Bayes classifier integrating perioperative clinical variables with 21-gene cardiovascular pharmacogenetic testing in 576 patients undergoing CABG, achieving accuracies of 0.81 in the test cohort and 0.79 in an independent validation cohort. Key contributors included ≥3-vessel CABG, heart failure, and the *KCNJ11* rs5219 polymorphism in combination with prolonged cardiopulmonary bypass time. SHAP analysis supported feature-level interpretability, and the model was deployed as a Web-based tool for individualized POAF risk estimation.

Collectively, these findings illustrate how multimodal ML frameworks can integrate routine clinical variables with emerging genomic and molecular biomarkers to improve perioperative risk stratification. However, prospective validation is required before these models can be reliably integrated into clinical workflows.

Taken together, these findings support a consistent predictive hierarchy across studies of POAF prediction. Demographic parameters and structural predictors, such as age, reflect a large portion of the variability in postoperative risk. Subsequent models incorporate perioperative and intraoperative variables, in combination with inflammatory markers, which may improve predictive performance. Optimal performance in the derivation cohort was achieved by ensemble approaches, particularly gradient-boosting frameworks that combine multiple high-performing models optimized on larger cohorts. That being said, model performance was attenuated in smaller validation cohorts, highlighting potential limitations in generalizability.

### Biochemical, metabolic, and stress-related biomarkers

In addition to clinical predictors, metabolic and biochemical markers provide insight into the physiologic stress response associated with cardiac surgery.

Cao et al^19^ identified the stress hyperglycemia ratio (SHR) as a predictor of POAF in a cohort of 3498 patients undergoing CABG from the Medical Information Mart for Intensive Care database. SHR, calculated as admission glucose divided by estimated average glucose, independently predicted POAF development, and ML models achieved an AUC of 0.813. Removal of SHR from the model reduced predictive performance, highlighting the importance of relative hyperglycemia as a marker of perioperative metabolic stress.

Akbulut et al^20^ used ML methods in a proof-of-concept study of 100 patients undergoing CABG and identified reduced serum magnesium, elevated total iron-binding capacity, and hypoalbuminemia as associated with increased arrhythmia risk. Although mechanistically intriguing, these findings are subject to cautious interpretation, given the limited sample size and lack of external validation.

Metabolomic profiling has also revealed novel predictive signatures. Yang et al^17^ identified 9 metabolites within pericardial fluid and serum that differentiated patients with POAF from non-POAF patients and constructed a 4-metabolite model capable of predicting postoperative arrhythmia risk. This provides a potentially exploratory metabolic fingerprint that necessitates external validation. However, like other targeted biochemical investigations, this metabolic fingerprint remains investigative and hypothetical at best rather than a clinically actionable predictive tool.

Collectively, these studies demonstrate that the strongest biochemical predictors of POAF reflect acute perioperative physiologic stress. These are mainly reflected by the degree of glycemic excursion, electrolyte depletion, and systemic inflammatory activation. Notably, similar structural and inflammatory determinants have also been identified in clinical ML models,^11^, ^12^, ^13^ suggesting a shared underlying pathophysiological substrate. However, replication in larger, prospectively validated cohorts remains necessary before clinical application.

### Anticoagulation considerations

Unlike chronic nonvalvular atrial fibrillation, POAF is often transient and occurs in the context of increased postoperative bleeding risk. Observational data suggest that long-term stroke risk after POAF is elevated but generally lower than that of persistent atrial fibrillation.^22^ AI-based prediction models may therefore support a 2-stage clinical framework by identifying patients at risk for POAF occurrence and predicting those likely to develop persistent or recurrent arrhythmia after discharge. Such risk stratification may inform decisions regarding anticoagulation therapy and long-term rhythm monitoring.

### Molecular and microRNA-based ML approaches

MicroRNAs (miRNAs) are epigenetic regulators of atrial remodeling, fibrosis, and electrophysiologic reprogramming and represent an emerging, yet preliminary, class of circulating molecular biomarkers for POAF prediction and risk stratification.

The molecular boundary encompasses miRNAs that serve as crucial markers for electrical remodeling and inflammatory processes. 1 study explored miRNA signatures in blood plasma to identify indicators of POAF using array-based profiling and advanced ML techniques, including Random Forest, XGBoost, Support Vector Machine, and K-Nearest Neighbors algorithms.^21^

The XGBoost model demonstrated an AUC of 0.83, in contrast to the Random Forest model, which attained 0.76, though findings are substantially limited by the very small sample (N = 15; 7 POAF vs 8 controls), which precludes meaningful assessment of overfitting or external generalizability. The 4 miRNAs—miR-96-5p, miR-184, miR-17-3p, and miR-200-3p—constituted the most significant signature for POAF, establishing links between genetic markers and pathways related to cardiac fibrosis and electrophysiologic remodeling.^21^

Among the approaches discussed, miRNA-based models are still in the early stages. While they provide insights into epigenetic mechanisms underlying atrial fibrosis and electrophysiologic remodeling, their small sample size and lack of external validity limit their practical use. In contrast, models built on large-scale, broadly available clinical data demonstrate consistent and clinically applicable predictive performance, as discussed in clinical feature–based models.

### Integrative interpretation and emerging paradigm

Recent studies demonstrate that AI-based predictive models can substantially improve identification of patients at risk for POAF after CABG. Reported AUC values across models range from approximately 0.80 to 0.93, exceeding those observed with traditional clinical risk scores.^11^, ^12^, ^13^ Importantly, heterogeneity in outcome definitions and underreporting of calibration metrics often confound this comparison.

**Figure 1:**
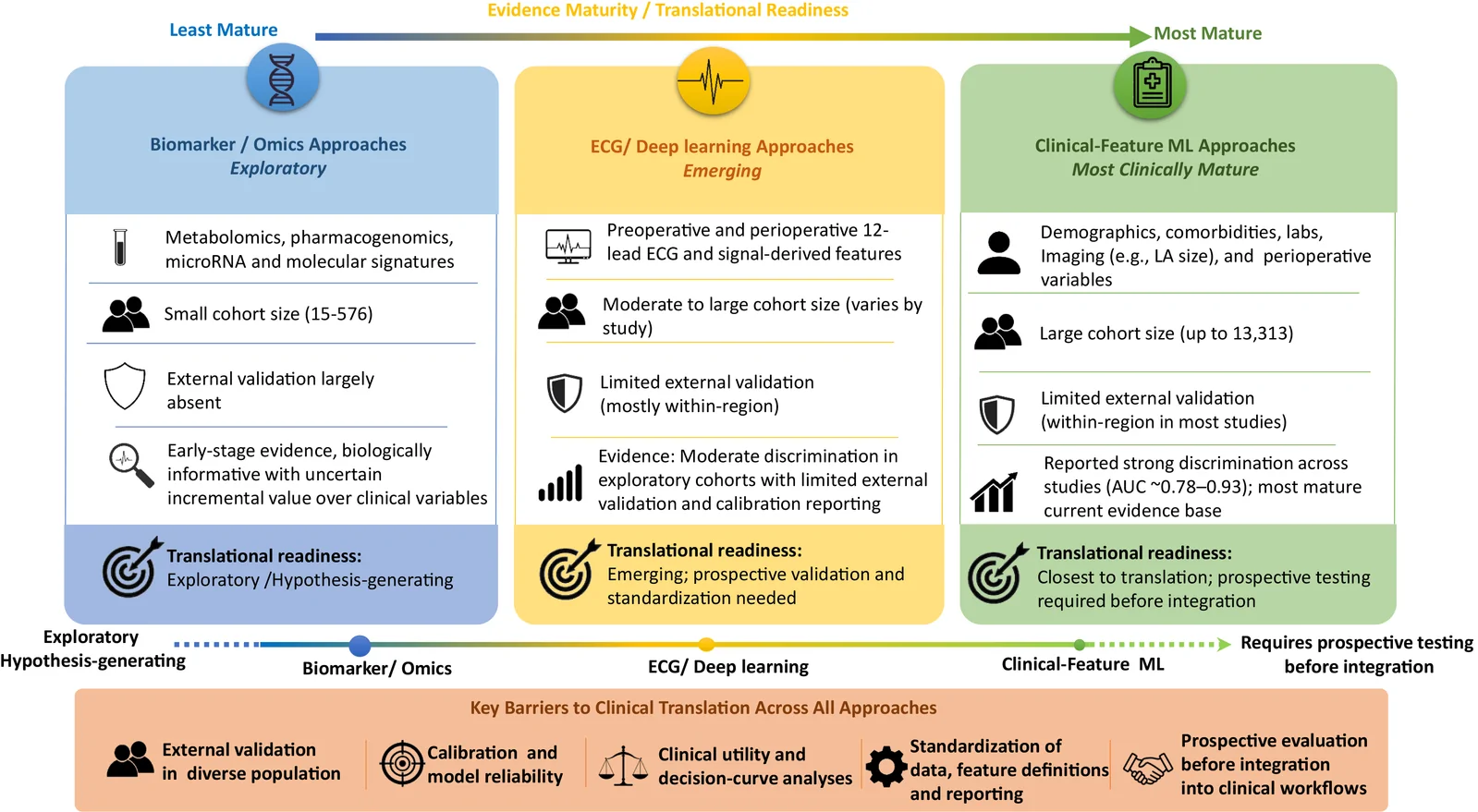
Evidence maturity and translational readiness of AI/ML modeling approaches for postoperative atrial fibrillation prediction after isolated coronary artery bypass grafting. The 3 AI/ML approaches synthesized in this review—biomarker/omics, ECG-based deep learning, and clinical-feature ML—are mapped across 4 indicators of evidence maturity: data inputs, cohort size, external validation, and current strength of evidence. The readiness spectrum ranks each approach from exploratory toward clinical translation. The bottom panel summarizes shared barriers to clinical translation. Positions reflect the evidence summarized in Table 1. No approach has yet undergone prospective validation. AI = artificial intelligence; AUC = area under the receiver operating characteristic curve; ECG = electrocardiography; LA = left atrial; ML = machine learning.

Performance can vary owing to several reasons, including sample size, feature space, method of validation, and outcome definition. The best-performing models shared common features, including larger derivation cohorts, use of ensemble methods or gradient boosting, and incorporation of external validation using independent data. Model performance often decreases with increasing data set heterogeneity and more rigorous external validation. This may be due to variability in factors affecting prediction such as surgical technique, preoperative prophylaxis strategies, and heterogeneity in patient phenotypes. From a maturity standpoint, externally validated models leveraging standard clinical and inflammatory data are the most developed for POAF prediction whereas omics-based approaches remain in early exploratory stages. The relative maturity of these AI/ML approaches across cohort size, external validation, and readiness for clinical translation is illustrated in Figure 1.

Integration of clinical variables with biochemical markers, genomic information, and electrocardiographic signals illustrates the multifactorial nature of POAF pathogenesis. Rather than representing a purely electrical disturbance, POAF appears to arise from a combination of metabolic stress, inflammatory activation, atrial structural remodeling, and genetic susceptibility. Explainable AI techniques further enhance the clinical utility of predictive models by identifying modifiable risk factors that may be targeted through perioperative management strategies.

While these models show promising translational direction, reported performance may be optimistic because of retrospective designs, single-center cohorts, and limited assessment of calibration and clinical utility. Importantly, SHAP analyses suggest that POAF predictive models may reliably identify key predictors such as age, atrial size, renal function, inflammatory markers, and cardiopulmonary bypass duration, irrespective of the underlying model architecture.

### Clinical implications

AI-based prediction models may have several potential clinical applications in perioperative cardiology. 1 potential application is predicting POAF through the development of a ML model integrated into electronic health record systems for real-time clinical decision support. An AI-based model using a panel of conventional clinical, laboratory, and electrocardiography-derived factors in conjunction with genomic information could robustly estimate for each patient an individualized risk for the development of POAF throughout the perioperative interval. With these estimates at hand, providers could then tailor their preventive measures (eg, use of statins and magnesium), optimize electrolyte and metabolic balances (such as potassium and glucose), and plan the intensity of postoperative surveillance.^5^^,^^6^

Another potential application relates to perioperative prophylaxis. A significant number of perioperative complications encountered in cardiology relate to acute inflammatory reactions. AI-based algorithms using relevant parameters may allow more accurate clinical predictions, thereby enabling precise timing and application of interventions such as preventive medication using β-blockers, amiodarone, posterior pericardiotomy, and anti-inflammatory treatment.^5^^,^^6^ Indeed, using such models would make it possible to apply the benefits of preventive treatment only to patients in whom the likelihood of complication is high, thereby avoiding the adverse effects secondary to these treatments in lower-risk patients. These side effects are notably bradycardia, hypotension, proarrhythmic effects, and bleeding.

AI models may also guide postoperative monitoring strategies. AI-based risk models may help guide the intensity of monitoring required during the postoperative period. Patients who are deemed to be at high risk of developing POAF may benefit from an increased intensity of monitoring during their postoperative period, including, but not limited to, an increased duration of rhythm monitoring, telemetry, and ambulatory electrocardiography.^5^^,^^6^ Conversely, patients who are considered to be at low risk may require less monitoring, which may help reduce resource utilization.

Despite promising performance, several factors determine whether these tools can be adopted clinically. Even if predictive accuracy is high, clinical utility depends on several additional considerations. These include understanding how interpretable models can be developed, the need for external validation, robust clinical calibration, and defining the predictive thresholds that would affect clinical decision making. Beyond POAF occurrence, clinically relevant outcomes include arrhythmia burden, need for therapeutic intervention, and implications for long-term anticoagulation and rhythm monitoring.

### Limitations and future directions

While it is encouraging that AI models frequently report improved discrimination compared with clinical risk scores for predicting patient outcomes, several important limitations must be considered when interpreting these findings. Most studies emphasize discrimination metrics such as AUC, whereas calibration, which assesses agreement between predicted risk and observed risk, as well as measures of clinical usefulness, such as decision curve analysis, is often inadequately reported, despite being critical for safe clinical implementation.^23^^,^^24^ In addition, the application of high-dimensional model architectures on relatively small patient cohorts introduces a substantial risk of model overfitting and may limit generalizability. Among all contemporary ML models discussed in this review, the majority report robust internal validation; external validation across diverse patient populations remains limited, limiting generalizability. As a result, reported model performance may be overestimated, particularly in single-center or geographically homogeneous data sets.

Furthermore, the apparent superiority of current models for POAF prediction is likely to be influenced by heterogeneity in study design, differences in patient population, outcome definition, time of data collection, and variability in institutional surveillance methods. Key methodological caveats include data leakage regarding timing of predictors and underexploration of important surgical factors such as on- vs off-pump technique and cross-clamp duration. These factors limit direct comparability between studies and complicate interpretation of relative model performance. In addition, sample sizes in molecular and biomarker-based studies remain relatively small, limiting the generalizability of results and suggesting that these approaches remain investigative rather than ready for clinical use. Finally, we focused this review on isolated CABG and did not include valve or combined cardiac surgery populations, which carry a higher incidence of POAF and may represent important settings for future AI-based risk prediction. Whether the modeling frameworks reviewed here transfer to these populations cannot be inferred from the present evidence base and should be addressed in dedicated, surgery-specific analyses.

Despite these limitations, AI-based prediction models remain a promising adjunct to clinical decision support for perioperative risk stratification of POAF. Future research should focus on rigorous prospective validation of these tools and evaluation of whether risk-guided preventive strategies reduce POAF incidence and associated complications and meaningfully influence clinical decision making. Integration of continuous physiologic monitoring data, such as wearable devices and telemetry, may also enable dynamic real-time prediction of arrhythmia risk.

## Conclusion

AI is increasingly being applied to predict POAF after CABG. ML models integrating clinical data with biochemical markers, pharmacogenomic information, and molecular biomarkers demonstrate improved predictive performance compared with traditional risk scores. As these technologies continue to evolve, prospective validation and rigorous calibration are imperative before clinical deployment. If these limitations are addressed robustly, AI-driven prediction tools may enable personalized perioperative risk stratification and targeted prevention strategies for patients undergoing cardiac surgery.

## Funding Sources

This research did not receive any specific grant from funding agencies in the public, commercial, or not-for-profit sectors.

## Authorship

All authors attest they meet the current ICMJE criteria for authorship.

## Disclosures

The authors have no conflicts of interest to disclose.
